# Safety of mapping in the sinus of valsalva region under intracardiac echocardiography guidance without angiography

**DOI:** 10.1016/j.ipej.2021.02.003

**Published:** 2021-02-08

**Authors:** Mike Al Asmar, Mayssam Houssari, Kinan Carlos El-Tallawi, Toufic Feghali, Marwan Refaat, Maurice Khoury, Bernard Abi-Saleh

**Affiliations:** American University of Beirut Medical Center, Beirut, Lebanon

**Keywords:** Arrhythmia, Aortic cusp, Catheter ablation, Intracardiac echocardiography, Premature ventricular contractions

## Abstract

**Background:**

Radiofrequency ablation at the region of the sinus of Valsalva carries a risk to the ostia of the coronary arteries. Coronary angiography is usually utilized to document a safe distance for mapping and ablation.

**Objective:**

To show that catheter ablation in the aortic root could be guided by phased-array intra cardiac echocardiography (ICE) and electro anatomic mapping without the need for coronary angiography.

**Methods:**

We reviewed all patients referred to our lab that underwent mapping and/or ablation in the sinus of Valsalva region. Procedures were carried out by operators that are skilled in the use of ICE. The need for angiography was documented, also the rate of success along with the immediate and 30-day complications rate.

**Results:**

Seventy patients (average age 48.7 ± 13.8 years; 64.3% males) were referred for ablation of ventricular and atrial arrhythmias. PVC constituted 95.7% of the cases. All patients underwent mapping and/or ablation at the sinus of Valsalva region without the need for coronary angiography to visualize the coronary ostia. Acute and effective ablation was achieved in 57 out of 70 (81.4%) patients partially effective ablation was achieved in 10 (14.3%) patients, and failure to ablate in the remaining 3 patients (4.3%). There was no occurrence of any adverse events, neither immediately or at day 30 after the procedure.

**Conclusion:**

In the hands of experienced operators, mapping and radiofrequency ablation in the sinus of Valsalva can be safely and reliably performed using intracardiac echocardiography alone without the need for supplementary catheter coronary angiography.

## 1 Introduction

Radiofrequency (RF) catheter ablation has proven to be a reliable and safe procedure for the treatment of both ventricular and supraventricular arrhythmias (SVT) [[1], [2], [3]]. However, when it comes to mapping and ablation in certain anatomic regions of the heart, the risk of complications becomes substantially high, warranting the use of specific modalities which would allow increased visualization of the catheter tip’s location with respect to different heart structures. Among such regions is the sinus of Valsalva (SoV) which is known to harbor critical structures like the coronary ostia, the aortic cusps, and the bundle of His. In addition to the SoV, mapping or ablation in the left and right ventricular outflow tracts (LVOT and RVOT, respectively) also carries a risk of damage to the conduction system. Finally, RF energy delivery against any cardiac surface should be of an inherent risk of thrombus formation at the ablation interface with subsequent risk of systemic embolization [4].

Since the coronary ostia are the major structures at risk during mapping and ablation at the level of the aortic cusps, current practice warrants the use of catheter coronary angiography to localize the origin of the coronary arteries relative to the ablation catheter’s tip [5]. However, invasive coronary angiography carries its own risks of complications.

Intracardiac echocardiography is a non-fluoroscopic imaging modality that enhances catheter visualization and navigation by providing continuous real-time imaging of intracardiac structures [5,6]. It was shown to enhance the safety and efficacy of ventricular arrhythmias ablation, especially in the outflow tracts, by providing a more accurate localization of the coronary ostia relative to the mapping catheter [6]. Moreover, ICE can aid in early detection and identification of procedural complications such as wall perforation and acute thrombi formation [4,7,8]. Therefore, we thought to assess the safety of mapping under intracardiac echocardiography (ICE) as a means of localizing the ablation catheter’s tip or ablating in these high risk locations without the need of performing a coronary angiogram.

## 2 Methods

We performed a retrospective chart review at our institution (American University of Beirut Medical Center, Beirut, Lebanon) of all patients who underwent mappings or ablations at the region of the sinus of Valsalva between June 2012 and December 2019. Procedures were carried out by operators skilled in the use of ICE. The need for angiography was documented, along with the immediate and 30-day complications rate. Acute success was defined as elimination of the predominant arrhythmia without recurrence for 12 h post ablation. Acute failure was defined as recurrence of the predominant arrhythmia within 12 h post ablation or inability to locate and ablate the region of interest. Effective ablation was defined as a reduction in arrhythmia burden by at least 80% either with or without anti-arrhythmic medication at 30 days of follow-up. Partially effective ablation was defined as a decrease in arrhythmia burden by 50–80% at 30 days of follow-up.

All cases included underwent mapping (but not necessarily ablation) in the region of the sinus of Valsalva. The site of successful ablation was labelled as the origin of the arrhythmia.

## 3 Electrophysiology study

Catheter selection and mapping systems were dependent on the operators. Electroanatomic mapping (NavX, St Jude Medical, St Paul, MN or Carto, Biosense Webster, Diamond Bar, CA) and phased-array ICE (St Jude Medical, St Paul, MN) were used to guide aortic cusp ablation in all cases. A retrograde aortic approach to map and ablate in the aortic cusps was used. Heparin was given to all patients after accessing the femoral artery. Activated clotting time was maintained between 200 and 300 s. A 3.5 mm open-irrigated catheter (Thermo CoolCelsius® RF ablation catheter) or a 4 mm diffuse open irrigated catheter (Therapy™ Cool Flex™ RF ablation catheter) were used for mapping and ablation. Radiofrequency energy was delivered in the “power-controlled” mode as follows: 20–30 W in the cusps and 35 to 45 below the cusps for 60–90 s depending on assessment of contact by ICE.

## 4 ICE views

**Fig. 1:**
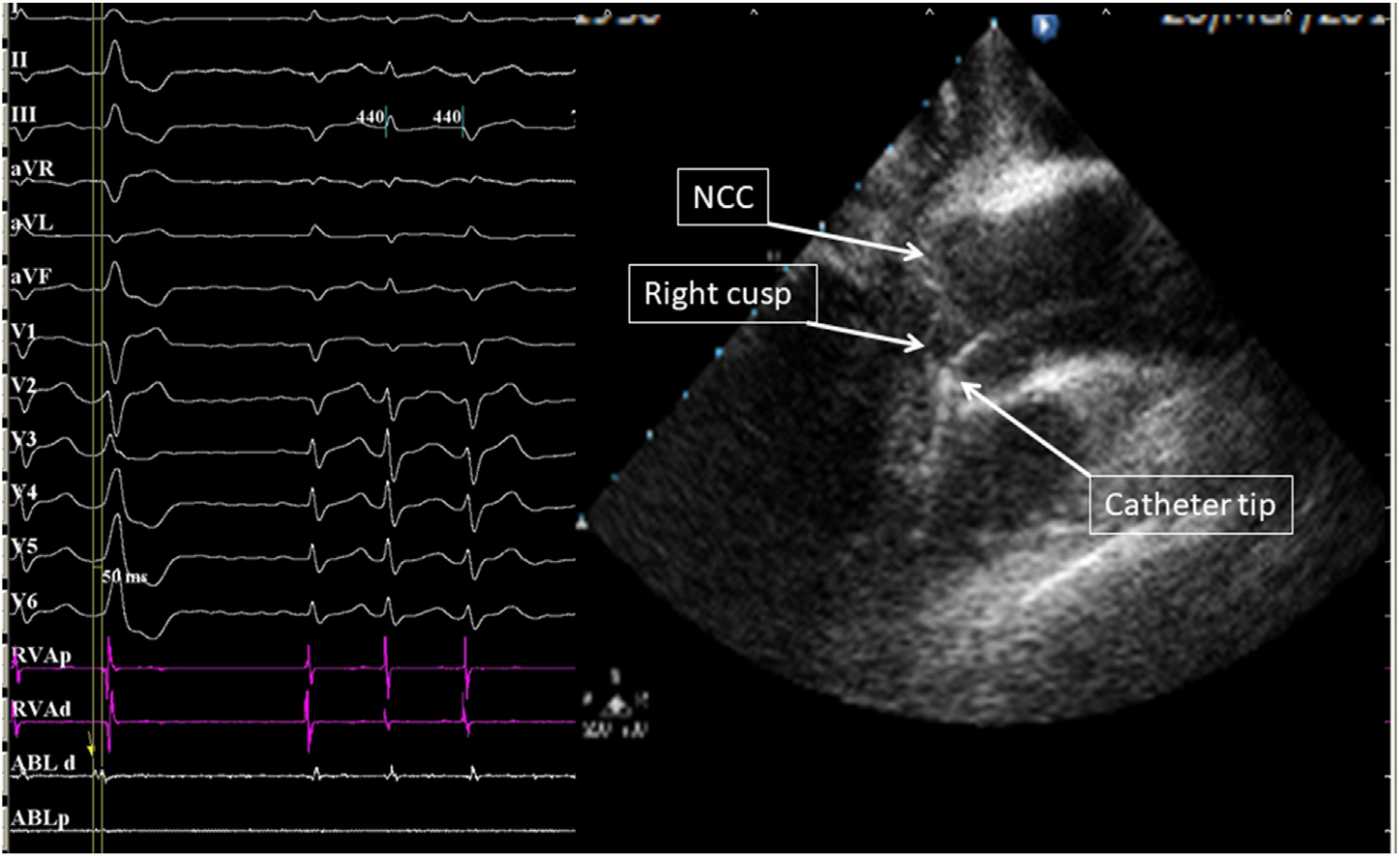
**Ablation from right coronary cusp.** Left panel: 12-lead surface electrocardiogram (ECG) of the premature ventricular contraction (PVC) originating from the right coronary cusp, along with signals originating from the right ventricle (RV) and ablation (ABL) catheters. The PVC signal on the distal ablation catheter is very early (50 ms) when compared to the surface ECG. On the right panel the ablation catheter is clearly visualized under ICE in direct contact with the right cusp (retrograde aortic approach), confirming the origin of the PVC at that location. NCC (non coronary cusp).

**Fig. 2:**
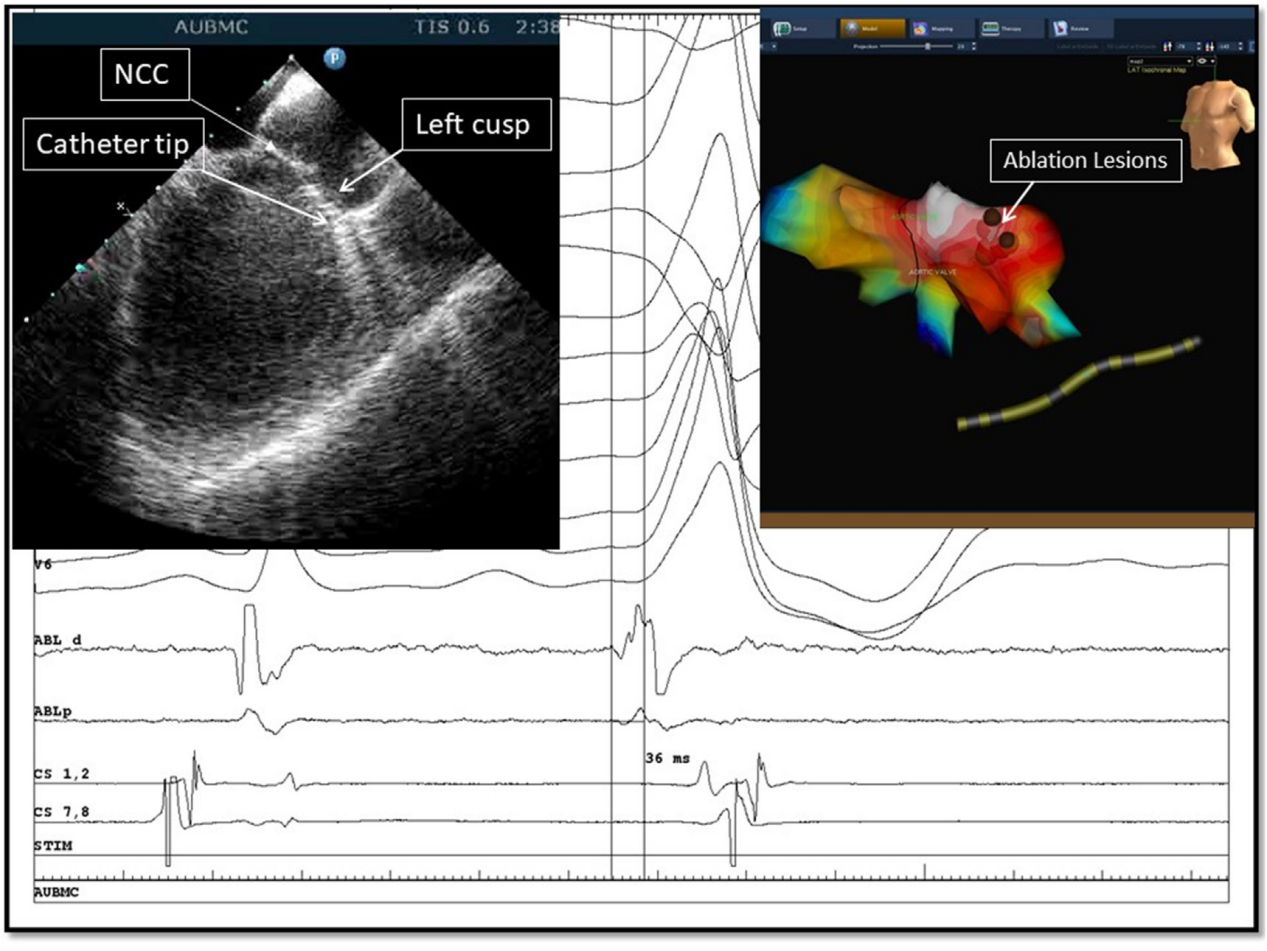
**Ablation from left coronary cusp.** A: Intracardiac echocardiogram view of the ablation catheter tip at the left coronary cusp. B: 12-lead surface ECG of the PVC originating from the left coronary cusp (LCC), along with depiction from the distal ablation (ABL) catheter showing a very early (36 ms) signal when compared to the surface ECG confirming a LCC origin. C: Electroanatomic map in left anterior oblique view illustrating the aortic valve along with the location of the ablation lesions (brown dots) over the left coronary cusp. NCC (non coronary cusp).

For mapping and/or ablation in the aortic cusps or below the cusps as well as the mitral annulus, the ICE catheter was positioned either in the right atrium or in the right ventricular inflow region, just past the tricuspid valve. The aortic valve was visualized in long axis and short axis. For ablations performed in the right or the left cusp, the left main and right coronary ostia were both visualized and at least a 1 cm distance was ensured between the catheter tip and the coronary ostium in the long axis view (Fig. 1). During mapping and/or ablation below the cusp, stable catheter position was confirmed in the long axis view (Fig. 2).

## 5 Results

Seventy patients (average age 48.7 ± 13.8 years; 64.3% males) were referred to our institution for mapping and/or ablation of ventricular and atrial arrhythmias in the region of the sinus of Valsalva. Premature ventricular tachycardias (PVC) constituted 95.7% of the cases, while the remaining (4.3%) were SVTs (Two atrial tachycardias and one concealed accessory pathway). We had to perform coronary angiography in 3 (4.3%) cases only. Two of these indications for angiography were chest pain occurring immediately following the ablation procedure (coronary angiography was normal in both cases). In the third case, angiography had to be performed before RF delivery at the anterior interventricular vein/great cardiac vein (AIV/GCV) junction in order to assess the proximity to the circumflex artery ostium. None of the cases required coronary angiography to visualize the ostia of the coronaries.

Acute and effective ablation was achieved in 57 out of 70 (81.4%) patients, partially effective ablation was achieved in 10 (14.3%) patients, and failure to ablate occurred in the remaining 3 patients (4.3%) due to proximity of the PVC earliest signal to his bundle with immediate induction of accelerated junctional rhythm upon brief energy delivery.

The ablation locations were: Left coronary cusp (LCC) 20 (28.6%), right coronary cusp (RCC) 18 (25.7%), non-coronary cusp (NCC) 4 (5.7%), mitral annulus 6 (8.6%), AMC 11 (15.7%), RVOT 3 (4.3%), LVOT 2 (2.9%), close to HIS location 2 (2.9%) and LV summit 7 (10%) (Table 1). There was no occurrence of any adverse events, neither immediately nor at day 30 after the procedure.

**Table 1:** Patients characteristics.

Study population	N = 70
Age	48.7 ± 13.8
Male gender	45 (64.3%)
Angiography	3 (4.3%)
PVC	67 (95.7%)
Atrial tachycardia	2 (2.9%)
Accessory Pathway	1 (1.4%)
RCC	18 (25.7%)
LCC	20 (28.6%)
NCC	4 (5.7%)
AMC	11 (15.7%)
RVOT	3 (4.3%)
LVOT	2 (2.9%)
MA	6 (8.6%)
LV Summit	7 (10%)
His	2 (2.9%)

AMC: aortic mitral continuity; LCC: left coronary cusp; LV: left ventricle; LVOT: left ventricular outflow tract; MA: mitral annulus; NCC: non-coronary cusp; PVC: premature ventricular contraction; RCC: right coronary cusp; RVOT: right ventricular outflow tract.

## 6 Discussion

Intracardiac echocardiography seems like an attractive modality for routine use in procedures involving mapping and ablation in the sinus of Valsalva region, while reserving catheter coronary angiography for cases with suboptimal ICE images. This is especially more appealing when the risks of coronary angiography are also considered. These include arterial vascular injury, contrast induced nephropathy, additional radiation exposure, and rarely coronary injury.

Hoffmayer et al. investigated the feasibility of ablation in the aortic root using ICE while keeping coronary angiography as a backup only when ICE views were not adequate [9].

Our experience, on a larger sample, confirms that this practice can be safely and successfully performed in the majority (95%) of the patients without the need of coronary angiography with no acute or long term adverse events. Importantly, angiography was done in two cases because of chest pain during the procedure and in one case for better coronary visualization of the circumflex artery since we had to ablate case in the coronary sinus. Therefore, none our cases required angiography to visualize the coronary ostia.

We find ICE as a highly valuable tool to visualize the NCC as well especially when targeting accessory pathways [10]. This cusp is recognized as the cusp facing the atria.

To our knowledge, this is the largest case series reporting the safety of mapping the sinus of Valsalva without the need for coronary angiography. Not a single case required coronary angiography in the purpose to visualize the coronary ostia. In all cases the ostia were well identified in one or two orthogonal ICE views.

## 7 Study limitations

The first limitations are that this is a retrospective case series that calls for a prospective randomized trial in order to confirm the results.

This is a single-center study, which could limit the generalizability of our results, and also mainly executed by experienced operator highly proficient in the use of ICE, comfortable in confirming a safe distance of the ablation catheter tip from certain critical heart structures. Therefore, this data cannot be generalized to operators with modest experience in ICE hence, in case of suboptimal ICE images we still recommend the utilization of coronary angiography in order to ensure safe distance of 10 mm from the coronary ostia. In addition, in the case of anomalous origin of coronary arteries ICE views might not clearly visualize the ostia and coronary angiography should be resorted to. No cases of anomalous origin of coronary arteries were seen in this case series.

Note that we included in our study cases not only cases that required ablation in the region of the SoV but also cases of only mapping in the SoV region and not necessarily requiring ablation there.

## 8 Conclusion

Our study suggests that, in the hands of experienced operators, mapping and radiofrequency ablation in the sinus of Valsalva can be safely and reliably performed under intra cardiac echocardiography guidance without the need for supplementary catheter coronary angiography.

## Disclosure

None of the authors have anything to disclose.

## Declaration of competing interest

We can also state that there is no financial arrangement or other relationship that could be construed as a conflict of interest.
